# Supplementary material for: Leveraging reddit data for context-enhanced synthetic health data generation to identify low self esteem
Source: Front Psychiatry. 2026 Jan 14;16:1726100. doi: 10.3389/fpsyt.2025.1726100 (PMC12847414; doi:10.3389/fpsyt.2025.1726100)
Supplement: Supplementary file 1 [file Table1.docx]

Appendix A: Sample of synthetic clinical note generated from a given Reddit post.

Reddit post

Everything's been all over the place lately and I feel i have no future. I've been looking out my balcony, just wanting to run and jump out but Im only at the seventh floor so like only broken bones. Overall, i don’t wanna wake up tomorrow.

Synthetic Clinical Note in SOAP Format

Subjective: The patient presented with feelings of hopelessness and despair, indicating a sense of having no future. The patient expressed suicidal ideation, stating a desire to end their life by jumping from the seventh-floor balcony. They mentioned that they don't want to wake up tomorrow, indicating a severe crisis. The patient's statement, "I feel I have no future," is a crucial insight into their mental state and a potential root cause of their distress. The patient's feelings of overwhelm and lack of purpose are further exacerbated by their current situation, suggesting a sense of desperation and loss of control. The patient's desire to escape their problems through self-harm is a common coping mechanism in individuals experiencing severe emotional pain.

Objective: The patient is a [insert age]-year-old individual presenting with suicidal ideation and feelings of hopelessness. The patient's statement, "I feel I have no future," is a clear indicator of their mental state and a potential root cause of their distress. The patient's suicidal ideation is characterized by a desire to end their life by jumping from the seventh-floor balcony. The patient's current mental state is severe, with a strong desire to escape their problems through self-harm.

Assessment: Based on the patient's presentation and statement, "I feel I have no future," it is clear that they are experiencing severe emotional distress. The patient's suicidal ideation and desire to escape their problems through self-harm are indicators of a severe mental health crisis. The patient's feelings of overwhelm and lack of purpose suggest a potential diagnosis of Major Depressive Disorder (MDD) or another mood disorder. The patient's statement, "I feel I have no future," is a critical insight into their mental state and a potential root cause of their distress. This statement suggests that the patient is experiencing a sense of hopelessness and despair, which is a common symptom of MDD.

Plan: 1. Immediate Intervention: The patient requires immediate attention and intervention to prevent harm to themselves. This may involve hospitalization or a crisis stabilization unit. 2. Mental Health Evaluation: A comprehensive mental health evaluation will be conducted to determine the underlying causes of the patient's distress and to establish a diagnosis. 3. Treatment Plan: A treatment plan will be developed in collaboration with the patient, their family, and mental health professionals. This plan will address the patient's suicidal ideation, feelings of hopelessness, and underlying mental health issues.

APPENDIX B: Engineered prompt for synthetic notes generation

| Prompt:  System instruction: In response to the patient’s notes and explanation, generate a short but structured clinical note in SOAP (Subjective, Objective, Assessment, Plan) format. Do not add, delete or update any new episodes or context. Make sure to include the explanation in clinical notes. Keep the length of clinical notes to 500 words.  User prompt: The patient’s notes is [NOTE] and the explanation is [CUES]. |
| --- |

APPENDIX C: Engineered prompt for identifying low self-esteem

| System Instruction: You are an expert in identifying episodes of low self-esteem from a given text. You are given a patient's note. Do not assume anything. Based only on the text, decide if the patient shows signs of LOW SELF-ESTEEM (1 = yes, 0 = no). Respond only in this format (1 or 0). Do not add any explanation. The note given is:  User prompt: I feel worthless most of the time and believe I am a burden to my family.  Assistant Response: 1 |
| --- |

APPENDIX D: Forward propagation in LoRA

Let the pre-trained weight matrix be $W_{0}=\mathbb{R}^{d\cdot k}$ where d and k are the number of rows and columns of the matrix whose entries are real numbers. In the standard fine-tuning, one would update the full matrix $W_{0}$. Instead, LoRA reduces the number of trainable parameters from ($d\cdot k$) to $r\left( d+k \right)$, and constrains the weight update $\Delta W=BA$to a low rank decomposition where $A\in\mathbb{R}^{r\cdot k}$ and $B\in\mathbb{R}^{d\cdot r}$, and $r\ll\min\left( d,k \right)$. Here, matrix A compresses the input $\left( k\to r \right)$ into a small subspace. and B expands it back (𝑟→𝑑) to match the original output size. The forward propagation then becomes

$h=W_{0}x+\frac{\alpha}{r}BAx$

where $\alpha$ is the scaling hyperparameter and $r$ is the rank. LoRA relies on a set of hyperparameters that jointly balance efficiency, stability, and adaptability.

APPENDIX E: Error analysis of low fidelity in synthetic notes generation.

| ID | Length of reddit post | Length of SHDG | Coverage rate | Context | Inferences | Relevant >0.3 |
| --- | --- | --- | --- | --- | --- | --- |
| 7 | 96 | 81 | 0.25 | Irrelevant* |  |  |
| 8 | 33 | 353 | 0.44 |  | Low self esteem | 1 |
| 15 | 42 | 386 | 0.5 |  | Hopelessness | 1 |
| 19 | 223 | 66 | 0.4 | Irrelevant* |  |  |
| 41 | 145 | 386 | 0 |  | Emotional distress | 0 |
| 43 | 40 | 369 | 0.44 |  | Self-criticism | 1 |
| 46 | 138 | 397 | 0 |  | Hopelessness | 0 |
| 48 | 20 | 89 | 0.25 | Irrelevant* |  |  |
| 63 | 174 | 52 | 0.37 | Irrelevant* |  |  |
| 67 | 43 | 65 | 0.25 | Irrelevant* |  |  |
| 90 | 208 | 131 | 0 | Irrelevant* |  |  |
| 92 | 29 | 42 | 0 | Irrelevant* |  |  |
| 96 | 144 | 387 | 0.4 |  | Self-criticism | 1 |
| 108 | 91 | 371 | 0.4 |  | Worthlessness | 1 |
| 110 | 207 | 69 | 0 | Irrelevant* |  |  |
| 132 | 89 | 51 | 0.4 | Irrelevant* |  |  |
| 154 | 184 | 323 | 0 |  | Self-criticism | 0 |
| 156 | 255 | 375 | 0.4 |  | Hopelessness | 1 |
| 161 | 41 | 338 | 0.44 |  | Self-destructive | 1 |
| 165 | 152 | 89 | 0.5 | Irrelevant* |  |  |
| 171 | 66 | 367 | 0.36 |  | Low self-esteem | 1 |
| 175 | 292 | 353 | 0.58 |  | Self-criticism | 1 |
| 192 | 254 | 359 | 0.4 |  | Self-criticism | 1 |
| 197 | 250 | 413 | 0.5 |  | Emotional distress | 1 |
| 201 | 160 | 18 | 0 | Irrelevant* |  |  |
| 210 | 147 | 368 | 0.25 |  | Low self esteem | 0 |
| 216 | 32 | 20 | 0 | Irrelevant* |  |  |
| 230 | 80 | 48 | 0.25 | Irrelevant* |  |  |
| 234 | 135 | 21 | 0.4 | Irrelevant* |  |  |
| 246 | 23 | 335 | 0 |  | Low self esteem | 0 |
| 247 | 46 | 69 | 0.5 | Irrelevant* |  |  |
| 270 | 94 | 394 | 0.2 |  | Emotional distress | 0 |
| 274 | 196 | 25 | 0 | Irrelevant* |  |  |
| 282 | 199 | 50 | 0.5 | Irrelevant* |  |  |
| 285 | 56 | 338 | 0.57 |  | Self-criticism | 1 |
| 287 | 203 | 91 | 0 | Irrelevant* |  |  |
| 292 | 91 | 386 | 0.55 |  | Hopelessness | 1 |
| 307 | 180 | 130 | 0 | Irrelevant* |  |  |
| 329 | 230 | 386 | 0.5 |  | Hopelessness | 1 |
| 343 | 171 | 78 | 0.33 | Irrelevant* |  |  |
| 346 | 258 | 402 | 0.44 |  | Hopelessness | 1 |
| 359 | 72 | 343 | 0.4 |  | worthlessness | 1 |
| 370 | 92 | 352 | 0.5 |  | burdensomeness | 1 |
| 376 | 105 | 57 | 0.01 | Irrelevant* |  |  |
| 416 | 87 | 125 | 0.5 | Irrelevant* |  |  |
| 428 | 83 | 65 | 0.5 | Irrelevant* |  |  |
| 439 | 80 | 103 | 0.25 | Irrelevant* |  |  |
| 445 | 145 | 115 | 0.4 | Irrelevant* |  |  |
| 451 | 42 | 381 | 0.5 |  | Self-destructive | 1 |
